# Spray-induced gene silencing enables the characterization of gene function during pre-penetration stages in *Blumeria graminis* f. sp. *tritici*

**DOI:** 10.3389/fpls.2025.1628068

**Published:** 2025-06-25

**Authors:** Meihui Zhang, Meijiao Zhou, Yunmeng Jia, Xipeng Li, Aolin Wang, Taiguo Liu, Yilin Zhou, Wei Liu, Jieru Fan

**Affiliations:** ^1^ State Key Laboratory for Biology of Plant Diseases and Insect Pests, Institute of Plant Protection, Chinese Academy of Agricultural Sciences, Beijing, China; ^2^ College of Plant Protection, Shenyang Agricultural University, Shenyang, China; ^3^ College of Agriculture and Forestry Science and Technology, Hebei North University, Zhangjiakou, China; ^4^ Heilongjiang Provincial Key Laboratory of Crop-Pest Interaction Biology and Ecological Control, Heilongjiang Bayi Agricultural University, Daqing, China

**Keywords:** Blumeria graminis f. sp. tritici, pre-penetration stage, spray-induced gene silencing, dsRNA, BgtActin

## Abstract

*Blumeria graminis* f. sp. *tritici* (*Bgt*), the causal agent of wheat powdery mildew, poses a significant threat to global wheat production. As an obligate biotroph, *Bgt* is recalcitrant to stable genetic manipulation. Although host-induced gene silencing has been used for gene function studies, it remains ineffective for targeting genes active during pre-penetration stages. Consequently, the functional roles of many *Bgt* genes during pre-penetration stages remain largely unexplored. In this study, the feasibility of spray-induced gene silencing (SIGS) to characterize gene function during pre-penetration stages was evaluated. The results demonstrated that *Bgt* conidia and germ tubes efficiently took up environmental double-stranded RNA (dsRNA), enabling the targeted silencing of *BgtActin*. Exogenous application of *BgtActin-dsRNA* effectively reduced target gene expression and impaired infection of *Bgt*. *BgtActin* silencing predominantly induced abnormal appressoria and reduced disease severity when dsRNA was applied at 6 and 10 hours post-inoculation (hpi). In contrast, *BgtActin* was almost not silenced when dsRNA was applied at 2 hpi. These findings established SIGS as a promising tool for gene functional studies during the pre-penetration stages of *Bgt* and highlight the potential of RNA-based strategies for the control of wheat powdery mildew.

## 1 Introduction

*Blumeria graminis* f. sp. *tritici* (*Bgt*) is the causal agent of wheat powdery mildew, a devastating disease that severely threatens wheat production and global food security (Singh et al., 2016; Jevtić et al., 2017). As an obligate biotroph, *Bgt* relies entirely on living host tissue for survival and reproduction. The conidia undergo a strictly programmed and highly synchronous asexual life cycle (Both et al., 2005). The infection process of *Bgt* begins with the emergence of a short primary germ tube approximately 30 minutes post-inoculation, which facilitates initial surface sensing and attachment (Yamaoka et al., 2006). Subsequently, an appressorial germ tube develops and differentiates into a swollen, hooked structure known as the appressorium. At 15 hours post-inoculation (hpi), a penetration peg forms beneath the appressorium, breaking both the host cuticle and cell wall by a combination of mechanical pressure and enzymatic degradation (Francis et al., 1996; Pryce-Jones et al., 1999). The peg does not breach the plant plasmalemma, and a haustorium develops in the periplasmatic space. These processes are completed at 24 hpi. Previous studies showed that when *B. graminis* exposed to high temperature, high humidity, or fungicide stress during pre-penetration stages, appressoria often were induced to be abnormal (Gilbert et al., 2009; Sugai et al., 2020; Wheeler et al., 2003; Zhang et al., 2022). These abnormal appressoria were caused by failure of the peg penetration into the host cells (Nonomura et al., 2010), which ultimately results in unsuccessful infection. However, the roles of key genes involved in pre-penetration stages remain largely unknown.

Host-induced gene silencing (HIGS) and virus-induced gene silencing (VIGS) have emerged as powerful tools for functional genomics in plant-pathogen interactions (Koch and Wassenegger, 2021; Nowara et al., 2010; Su et al., 2024; Zhang et al., 2023). These approaches leverage the host’s RNA interference (RNAi) machinery to deliver small RNAs into interacting fungi, inducing gene silencing in a cross-kingdom manner (Cai et al., 2018). However, the dependence of HIGS and VIGS on host-mediated RNAi limits their efficiencies in some cases for studying gene function. For example, these approaches are ineffective during the asymbiotic and presymbiotic stages of *Rhizophagus irregularis*, when direct exchange with the host has not yet been established (Fan et al., 2025). In *Bgt*, haustoria are essential for nutrient uptake and for the delivery of virulence effectors that suppress host defenses (Both et al., 2005; Bozkurt and Kamoun, 2020). However, during the pre-penetration stages, *Bgt* has not yet formed haustoria, and there is nearly material exchange between the pathogen and the wheat (Zhang et al., 2005). As a result, host-derived small RNAs are unlikely to be effectively delivered into *Bgt* cells at these stages, thereby limiting the efficiency of HIGS and VIGS.

Recent studies have shown that certain fungal pathogens can directly take up environmental double-stranded RNAs (dsRNAs) and initiate gene silencing through endogenous RNAi pathways, a strategy known as spray-induced gene silencing (SIGS, Koch et al., 2016; McRae et al., 2023; Ouyang et al., 2024; Qiao et al., 2021; Wang et al., 2016). The exogenous application of dsRNA or single-stranded RNAs onto fungal tissues has been shown to trigger the production of small RNAs that target essential fungal genes, thereby reducing infection and disease development (Koch et al., 2016; McRae et al., 2023; Ouyang et al., 2024; Qiao et al., 2021). However, whether *Bgt* can take up exogenous dsRNA with high efficiency to induce gene silencing is still unknown.

*Actin* is a highly conserved cytoskeletal protein and involves in nearly all fundamental eukaryotic cellular processes (Berepiki et al., 2011). In filamentous fungi, actin plays a pivotal role in cell morphogenesis and polarized growth (Walker and Garrill, 2006). In this study, SIGS was applied to functionally characterize *BgtActin*, aiming to explore the feasibility of SIGS during the pre-penetration stages of *Bgt*. We demonstrated that exogenous application of dsRNA via SIGS effectively silenced target gene expression, enabling functional analysis of *Bgt* genes during pre-penetration stages. Furthermore, our findings revealed that *BgtActin* was required for the penetration process, providing new insights into its role in *Bgt* establishment. These findings also lay the foundation for the development of RNA-based strategies for disease management targeting *Bgt*.

## 2 Materials and methods

### 2.1 Plant materials and inoculation

Seeds of the highly powdery mildew-susceptible wheat cultivar ‘Jingshuang16’ were sown in glass tubes covered with 5 layers of gauze to prevent accidental contamination and maintained in a climate-controlled growth chamber at 18°C ± 0.5°C, with a 16-h-light/8-h-dark cycle. At the one-leaf seedling stage, detached leaf segments were prepared by excising leaves into 3.5 cm segments, which were then placed on a water agar medium supplemented with 60 mg mL^-1^ benzimidazole to delay senescence. *Bgt* conidia were inoculated onto the leaf segments using a settling tower, followed by incubation at 18°C.

### 2.2 Transcriptomic analysis

To analyze the expression profiles of *BgtActin* during pre-penetration stages of *Bgt*, we examined published transcriptome deep sequencing (RNA-Seq) data. Raw RNA-Seq reads were downloaded from the NCBI Sequence Read Archive under the BioProject number PRJNA1237996 (Zhang et al., submitted)^1^. These reads were derived from *Bgt* isolates 13-14-7-2-2 and 13-14-8-2-2 collected at different infection time points (0, 15, 24, and 48hpi). The clean reads were mapped to the reference genome of *Bgt* isolate 96224 (GCA_900519115.1) (Müller et al., 2019) by HISAT2 (Kim et al., 2015). Gene expression levels were normalized using fragments per kilobase of transcript per million mapped reads (FPKM). Differential expression gene (DEG) analysis was performed using the DESeq2 package (Love et al., 2014) in RStudio (v4.2.1). DEGs were identified using the criteria the Benjamini-Hochberg adjusted *P* value ≤ 0.05, |log_2_ Fold Change| > 1.

### 2.3 dsRNA design and synthesis

**Figure 1:**
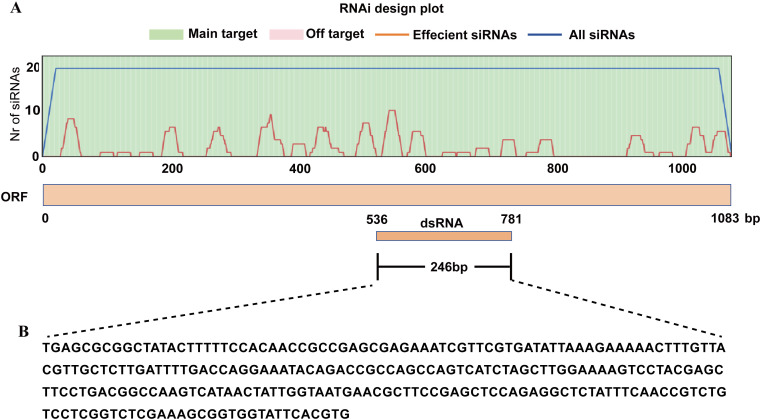
Design **(A)** and sequence **(B)** of dsRNA targeting *BgtActin* gene.

dsRNA was designed using the siRNA-Finder software (Lück et al., 2019) to minimize off-target effects in the host plant. Target region enriched with multiple high-efficiency small interfering RNA sites was selected for *BgtActin* (GenBank: VDB84307.1) to synthesize *BgtActin-dsRNA* (**Figure 1A**). The target sequence was 246 bp in length (**Figure 1B**). RNAi fragment targeting *BgtActin* was amplified from cDNA derived from *Bgt*-infected wheat leaves using gene-specific primers. Each primer included a T_7_ RNA polymerase promoter sequence (5’-TAATACGACTCACTATAGGG-3’) at both the 5’ and 3’ ends (**Table 1**). The dsRNAs were synthesized *in vitro* using the T_7_ RNAi Transcription Kit (Vazyme, Nanjing, China) and subsequently purified according to the manufacturer’s instructions. To investigate whether *Bgt* is capable of exogenous dsRNA uptake (Koch et al., 2016; Wang et al., 2016), a fluorescein-labeled *BgtActin-dsRNA* was generated by *in vitro* transcription with T_7_ RNAi Transcription Kit (Vazyme, Nanjing, China) in the presence of fluorescein-12-UTP (Sigma, Saint Louis, MO, USA), following the manufacturer’s protocols.

**Table 1:** Primers of RNAi fragments used in this study.

Primer name	Primer sequence (5’-3’)	Purpose
Actin_T_7_-F	taatacgactcactatagggTGAGCGCGGCTATACTTTTT	For the amplifing of RNAi fragments targeting *BgtActin*
Actin_T_7_-R	taatacgactcactatagggACGTGAATACCACCGCTTTC	
18SrRNA F	TAGTTGGTGGAGTGATTTGT	For real-time quantitative PCR
18SrRNA R	CGTTGGCTCTGTCAGTGTAG	
Actin-F_qpcr_	GGTTTCTCTCTTCCACACGC	
Actin-R_qpcr_	CACGAACGATTTCTCGCTC	

### 2.4 External application of dsRNA on the surface of detached leaf segments

To investigate the exogenous dsRNA uptake capability and the effect of gene silencing during pre-penetration stages, *Bgt* conidia were inoculated onto the detached leaf segments, then *BgtActin-dsRNA* and fluorescein-labeled *BgtActin-dsRNA* were sprayed onto inoculated leaf surfaces at 2, 6 and 10 hpi, respectively (**Supplementary Figure S1**). A total of 60 μg of dsRNA per treatment (at a concentration of 60ng/μL) was applied to inoculated leaves. Leaves sprayed with RNase-free water were used as the control.

### 2.5 RNA isolation and real-time quantitative PCR

To analyze gene expressions of *BgtActin*, samples were collected at 24, 48 and 72 hpi and snap frozen in liquid nitrogen. Total RNA was extracted from a frozen sample with TransZol Up Plus RNA Kit Trizol reagent (TRANSGEN BIOTECH, Beijing, China). The first-strand cDNA was synthesized with FastKing One-Step RT-PCR Kit (TIANGEN, Beijing, China). Real-time quantitative PCR amplifications were conducted with TranStart Top Green qPCR SuperMix (TRANSGEN BIOTECH, Beijing, China) and performed on the ABI 7500 real-time PCR system. Relative expressions were calculated using the 2^-△△CT^ method (Livak and Schmittgen, 2001) with 18S rRNA as the reference gene. Three biological replicates were performed for statistical analysis. The primers used for real-time quantitative PCR were listed in **Table 1**.

### 2.6 Staining and histological observation

To investigate the exogenous dsRNA uptake capability, the visualization via confocal microscopy was completed at 24 hpi. Leaves sprayed with *BgtActin-dsRNA* were treated with micrococcal nuclease (NEB, Ipswich, MA, USA) at 37°C for 30 min to remove surface-bound dsRNAs from *Bgt* conidia or hyphae. Leaves sprayed with RNase-free water were used as a control. The fluorescent signal was examined using a Zeiss LSM880 confocal microscopy. The excitation/emission wavelengths were 488/519 nm. Fluorescent image processing was performed using ZEN blue software.

The histological observation was conducted on a fluorescence microscope Olympus BX61. To assess conidia germination, over 200 conidia per leaf segment were randomly selected, and the number of conidia producing primary germ tubes was counted. Simultaneously, appressoria formation was evaluated by the records of both normal and abnormal appressoria. For each treatment, 3 leaf segments were examined, and the experiment was conducted with three biological replicates.

Leaf segments infected by *Bgt* isolate were stained with Coomassie blue solution. At 24 hpi, the leaf segments were fixed in a fixative (ethanol: acetic acid, 1: 1, v/v) for 24 hours, then bleached in a destaining solution (lactic acid: glycerol: H_2_O, 1: 1: 1, v/v) for 48 hours. Subsequently, the samples were stained for 10 minutes with 0.6% (w/v) Coomassie blue solution, followed by thorough rinsing with distilled water.

### 2.7 Data analysis

Two-way analysis of variance with SAS software version 9.4 (SAS Institute Inc., Cary, NC, USA) was used to assess the difference in conidia germination frequency, formation frequency of appressoria and abnormal appressoria, and disease severity between different treatments or timing of dsRNA application. One-way analysis of variance was used to test the effect on gene expression levels when *BgtActin*-*dsRNA* was applied.

## 3 Results

### 3.1 *Bgt* was capable of environmental dsRNA uptake

**Figure 2:**
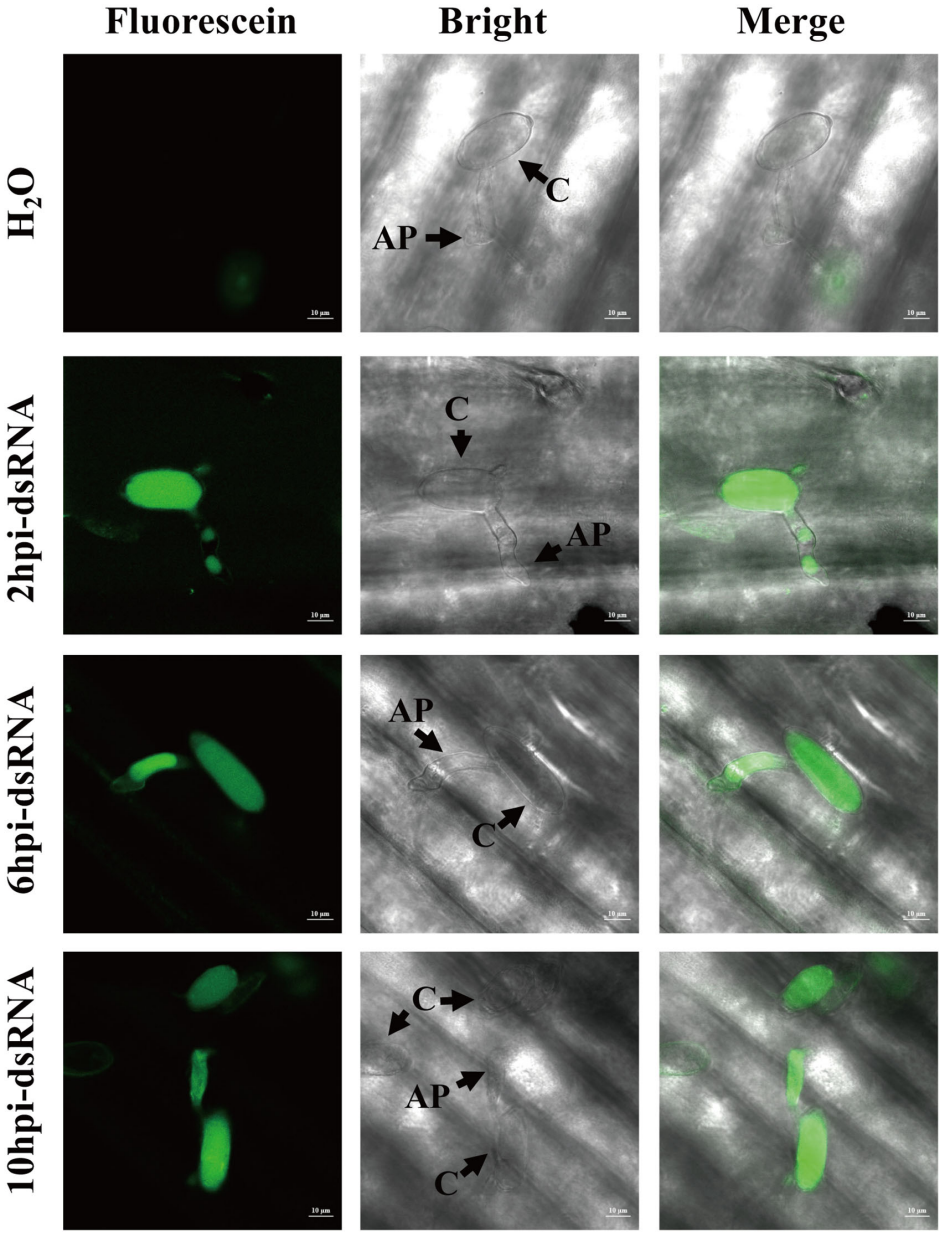
The efficiency of double-stranded RNA (dsRNA) uptake by *Blumeria graminis* f. sp. *tritici* using fluorescein-labeled dsRNA sprayed at 2, 6 and 10 hours post-inoculation (hpi). H_2_O was the control with spraying RNase-free water; “2hpi-dsRNA”, “6hpi-dsRNA” and “10hpi-dsRNA” were sprayed with *BgtActin*-*dsRNA* at 2, 6 and 10 hpi, respectively. The fluorescent signals of all treatments were observed via confocal microscopy at 24 hpi. C, conidium; AP, appressorium.

To evaluate the exogenous dsRNA uptake capability during pre-penetration stages, conidia were treated with fluorescein-labeled dsRNA at 2, 6 and 10 hpi. Fluorescent signals were clearly observed in *Bgt* cells at 24 dpi for all sprayed time, even after micrococcal nuclease treatment, while no fluorescein signal was detected in water-treated controls (**Figure 2**). Notably, dsRNA was evidently taken up during multiple pre-penetration stages, including conidia germination and appressoria formation. These results indicated that *Bgt* can directly take up environmental dsRNA, independent of the host plant.

### 3.2 Expression level of *BgtActin* after treated with *BgtActin*-*dsRNA*

**Figure 3:**
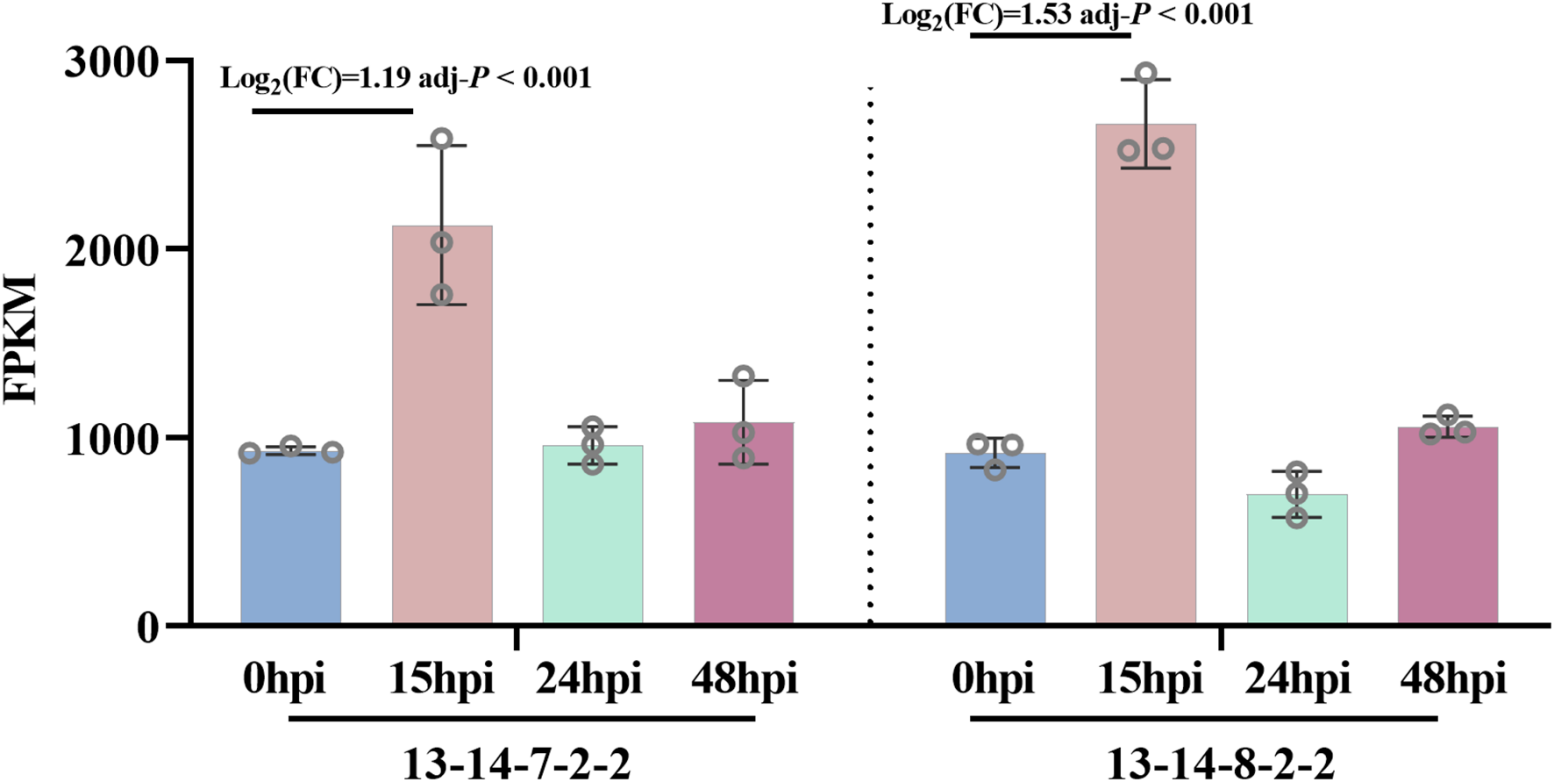
Expression levels of *BgtActin* in different isolates using RNA-seq data during early infection stages. Expression levels were indicated as FPKM (fragments per kilobase per million reads), log_2_FC (fold change) >1 and adj-*P* (Benjamini-Hochberg adjusted *P* value) ≤ 0.05 was considered significant.

**Figure 4:**
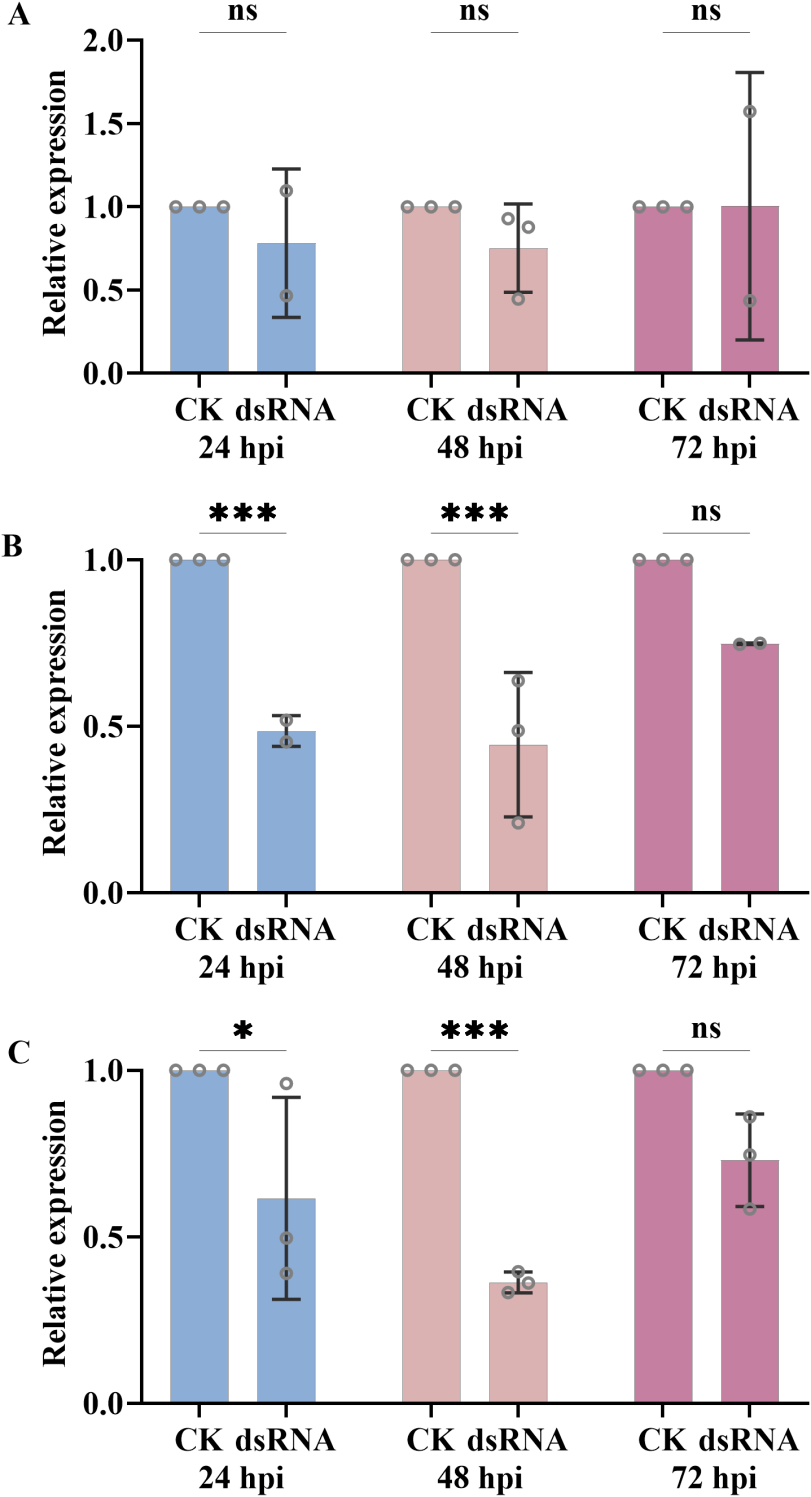
Relative expression levels of *BgtActin* in *Blumeria graminis* f. sp. *tritici* at 24, 48 and 72 hours post-inoculation (hpi) following the application of dsRNA. **(A)** application of dsRNA at 2 hpi; **(B)** application of dsRNA at 6 hpi; **(C)** application of dsRNA at 10 hpi. 18SrRNA was used as the reference gene. The error bar showed standard error of three biological replicates. Data were analyzed by one-way analysis of variance with Duncan’s multiple range test (ns *P* > 0.05, *0.01<*P* ≤ 0.05 and ***P ≤ 0.001).

Transcriptomic analysis showed that the expression levels of *BgtActin* peaked at 15 hpi in both tested isolates (13-14-8-2-2-2 and 13-14-7-2-2), showing a significant up-regulation compared with 0 hpi (**Figure 3**). In contrast, no significant differences in the expression levels of *BgtActin* were observed at 24 or 48 hpi compared with 0 hpi (**Figure 3**). Moreover, the expression levels of *BgtActin* at 24, 48 and 72 hpi were not significantly reduced when *BgtActin*-*dsRNA* were applied at 2 hpi, compared with the water-treated control (**Figure 4A**). However, the expression levels of *BgtActin* were significantly down-regulated at 24 and 48 hpi, while application of *BgtActin*-*dsRNA* at 6 and 10 hpi (**Figure 4B, C**). By 72 hpi, expression levels were no longer significantly different from the control, regardless of treatment timing (**Figures 4A–C**). These results suggested that *BgtActin* can be effectively silenced by spraying *BgtActin*-*dsRNA* during pre-penetration stages.

### 3.3 Effects of *BgtActin* silencing on pre-penetration stages of *Bgt*

**Figure 5:**
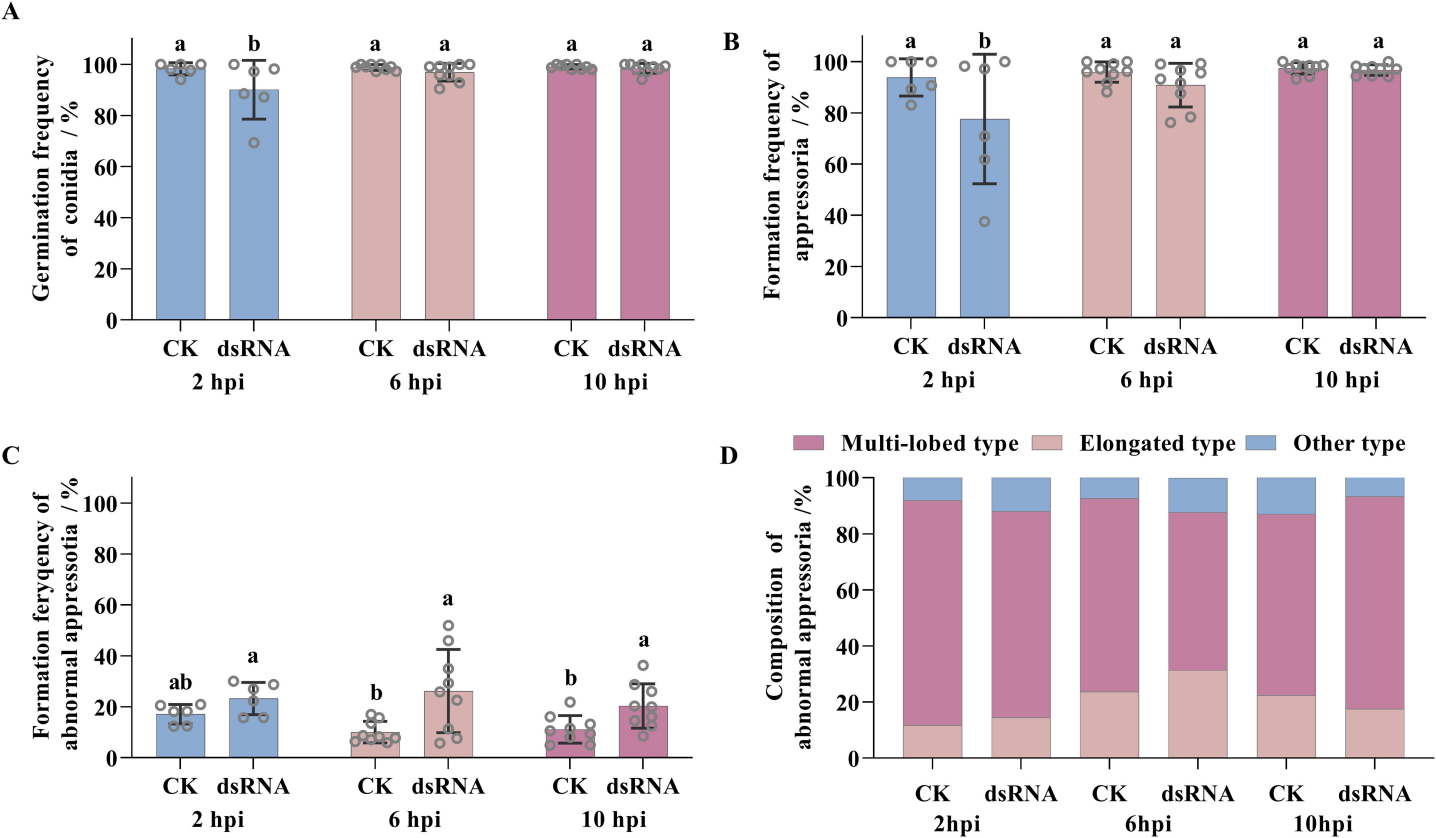
Effects of *BgtActin* silencing on germination frequencies of conidia **(A)**, formation frequencies of appressoria **(B)** and abnormal appressoria **(C)** of *Blumeria graminis* f. sp. *tritici*. **(D)** Composition of abnormal appressoria in different treatment. The error bar showed standard error of three biological replicates. Data were analyzed by two-way analysis of variance with Duncan’s multiple range test. The different letters above the error bars indicated significant differences (*P* ≤ 0.05).

**Figure 6:**
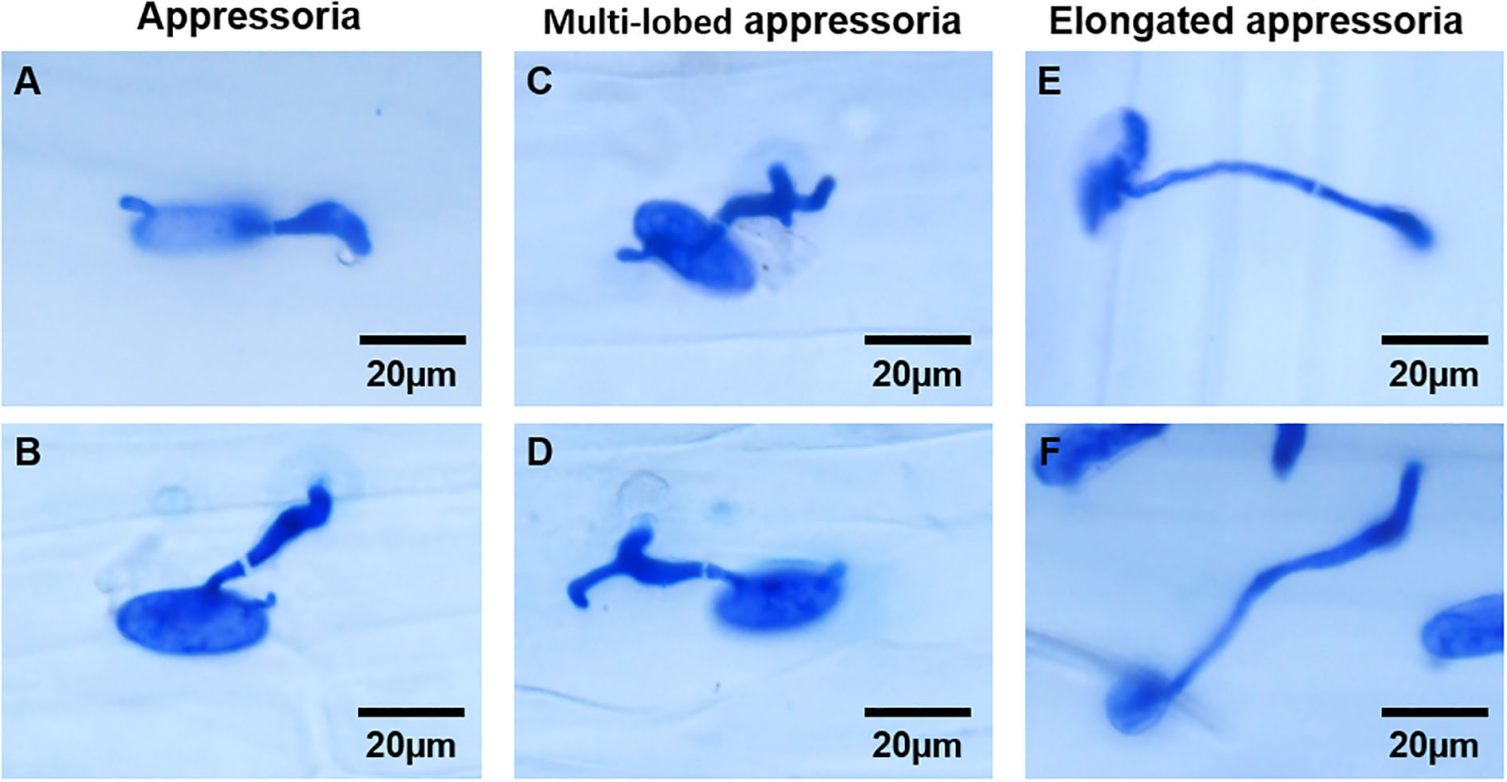
Normal **(A, B)** and abnormal appressoria **(C-F)** of *Blumeria graminis* f. sp. *tritici* at 24 hours post-inoculation.

The frequencies of conidia germination and appressoria formation in the *Bgt* isolate were significantly reduced when *BgtActin-dsRNA* was applied at 2 hpi, compared with the water-treated controls (**Figures 5A, B**; **Supplementary Table S1**). However, there were no significant differences in the frequencies of conidia germination and appressoria formation between *BgtActin-dsRNA* application (at 6 or 10 hpi) and water treatment (**Figures 5A, B**; **Supplementary Table S1**). Notably, the frequency of abnormal appressoria formation was significantly increased following *BgtActin*-*dsRNA* application at 6 or 10 hpi, compared to water-treated controls, suggesting interference with penetration peg formation (**Figure 5C**; **Supplementary Table S1**). Further histological analysis revealed that most abnormal structures exhibited multi-lobed appressoria (**Figures 5D**, **6**; **Supplementary Table S1**).

### 3.4 *BgtActin* silencing reduced disease severity of *Bgt* on wheat leaves

**Figure 7:**
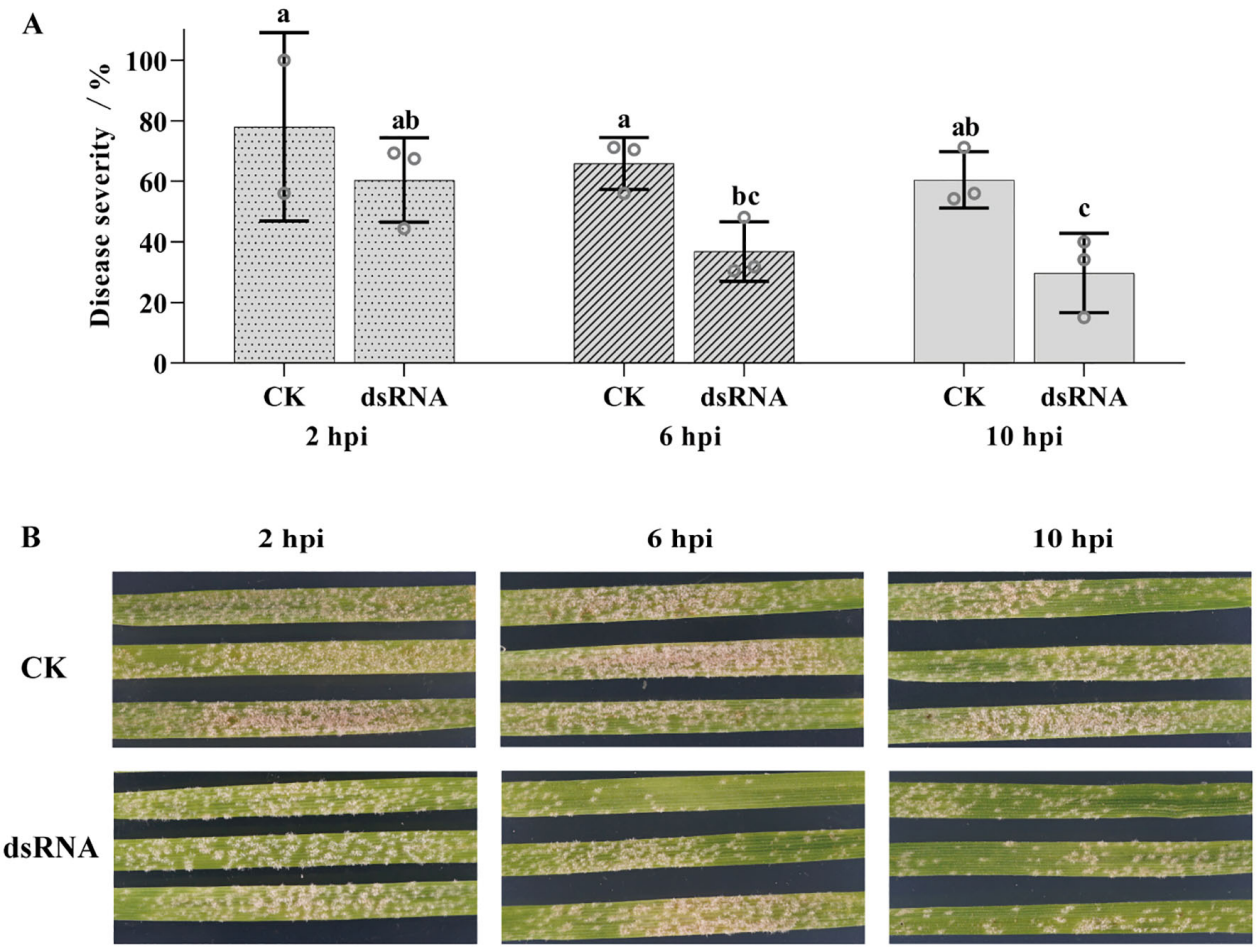
Treatment of double-stranded RNA (dsRNA) targeting *BgtActin* reduced *Blumeria graminis* f. sp. *tritici* infection. **(A)** The disease severity at 8 days post-inoculation (dpi) in different treatments. CK was the control with spraying RNase-free water; application of *BgtActin*-*dsRNA* was at 2, 6 and 10 hours post-inoculation (hpi), respectively. The error bar showed standard error of three biological replicates. Data were analyzed by two-way analysis of variance with Duncan’s multiple range test. The different letters above the error bars indicated significant differences (*P* ≤ 0.05). **(B)** Representative photographs of phenotypes at 8 dpi sprayed with RNase-free water and *BgtActin-dsRNA* at 2, 6 and 10 hpi.

Application of *BgtActin*-*dsRNA* at 6 or 10 hpi significantly reduced powdery mildew severity compared to the control, with inhibition proportions ranging from 26% to 50% (**Figure 7**). In contrast, no significant difference in disease severity was observed between *BgtActin*-*dsRNA* and control treatments when the dsRNA was applied at 2 hpi (**Figure 7**). These findings indicated that the effect of the exogenous dsRNA targeting *BgtActin* on inhibiting disease development depended on the timing of application.

#### 3.4.1 Key factor influencing *BgtActin* silencing

Variance analysis showed that both dsRNA treatment and application timing had significant effects on conidia germination, appressoria formation frequency, and disease severity (**Table 2**). Specifically, dsRNA treatment and application timing account for 41.4% and 24.2% of the total variance in disease severity, respectively. In contrast, the timing of application did not significantly affect the frequency of abnormal appressoria formation, whereas dsRNA treatment had a significant effect, accounting for 25.3% of the total variance in this phenotype (**Table 2**). Notably, the effect of interactions between application timing and dsRNA treatment was not statistically significant for any of the phenotypes assessed (**Table 2**).

**Table 2:** Two-factor variance analysis of application timing and dsRNA treatment targeting *BgtActin* in pre-penetration stages of *Blumeria graminis* f. sp. *tritici*.

Phenotype	Source of variation	DF	Sum Sq	Mean Sq	*F*-value	*P* (*>F*)
Germination frequency of conidia	Timing of application (A)	2	0.016	0.008	4.080	0.024
	dsRNA treatment (B)	1	0.015	0.015	7.520	0.009
	A*B	2	0.011	0.006	2.860	0.068
	Error	42	0.082	0.002		
	Total variation	47	0.124			
Formation frequency of appressoria	Timing of application (A)	2	0.094	0.047	4.640	0.015
	dsRNA treatment (B)	1	0.062	0.062	6.150	0.017
	A*B	2	0.045	0.022	2.200	0.123
	Error	42	0.425	0.010		
	Total variation	47	0.626			
Formation frequency of abnormal appressoria	Timing of application (A)	2	0.014	0.007	0.890	0.417
	dsRNA treatment (B)	1	0.127	0.127	15.740	<0.001
	A*B	2	0.020	0.010	1.240	0.299
	Error	42	0.340	0.008		
	Total variation	47	0.501			
Disease severity	Timing of application (A)	2	0.163	0.081	4.040	0.048
	dsRNA treatment (B)	1	0.277	0.277	13.770	0.003
	A*B	2	0.013	0.007	0.330	0.725
	Error	11	0.221	0.020		
	Total variation	16	0.674			

*P* ≤ 0.05 represent statistical significance.

## 4 Discussion

*Bgt* is an obligate biotrophic pathogen and undergoes a strictly programmed and highly synchronous asexual life cycle (Both et al., 2005). Its pre-penetration stages involve several critical steps, including conidia germination, appressorium formation and maturation, and penetration of the host epidermal cells. Each of these stages is critical for the successful establishment of infection. Among them, penetration of epidermal cells is particularly susceptible to environmental stresses and host immune responses (Gilbert et al., 2009; Sugai et al., 2020; Wheeler et al., 2003; Zhang et al., 2022). In *Magnaporthe oryzae*, the development and function of the appressorium have been extensively studied, revealing mechanisms involved in turgor generation, maturation, penetration, and transpressorium formation (Dagdas et al., 2012; Qi et al., 2018; Ryder et al., 2022; Yi and Valent, 2013). However, similar studies in *B. graminis* remain limited, primarily due to the difficulty of performing stable genetic transformations in this obligate biotroph.

In this study, we demonstrated that *Bgt* conidia and germ tubes are capable of directly absorbing exogenous dsRNA during pre-penetration stages, including conidia germination and appressorium formation (**Figure 2**). The absence of haustoria at these stages excludes the possibility of host-derived sRNA delivery, highlighting the importance of direct uptake. Similar dsRNA uptake capabilities have also been reported in other plant pathogenic fungi, such as *Botrytis cinerea*, *Fusarium graminearum*, *Golovinomyces orontii and Phakopsora pachyrhizi* (Koch et al., 2016; McRae et al., 2023; Ouyang et al., 2024; Qiao et al., 2021; Wang et al., 2016).

Leveraging this capability, we successfully silenced *BgtActin* using exogenous dsRNA during pre-penetration stages. Notably, in this study, the silencing efficiency was dependent on the timing of dsRNA application. Application at 6 or 10 hpi significantly reduced *BgtActin* expression, induced abnormal appressoria, and suppressed disease severity, whereas application at 2 hpi showed minimal effects. Although previous studies in other fungi demonstrated that exogenous dsRNA targeting genes such as *CYP51* (cytochrome P450 51) and *DCLs* (Dicer-like proteins) could effectively suppress pathogen development and virulence (Koch et al., 2016; McRae et al., 2023; Qiao et al., 2021; Wang et al., 2016), none have investigated the impact of application timing on silencing efficiency and disease control. Furthermore, while gene silencing and associated phenotypic effects were evident at 24 and 48 hpi, they diminished by 72 hpi (**Figure 4**), indicating a temporal limitation of SIGS, at least for *BgtActin*. Whether such temporal limitations are gene-specific remains to be clarified.

HIGS has been extensively used for gene functional studies in obligate biotrophs such as *Bgt* and *Puccinia striiformis* f. sp. *tritici* (Nowara et al., 2010; Qi et al., 2018). However, HIGS mainly targets genes expressed during haustorium formation and is therefore unsuitable for investigating genes acting during the pre-penetration stages, when the host-pathogen interface has yet formed. SIGS, based on the direct uptake of exogenous dsRNA by conidia and germ tubes of *Bgt*, effectively overcomes this limitation. Similar to HIGS, the efficiency of SIGS also depends on the functional importance of the targeted gene. Previous studies have shown that not all genes are suitable RNAi targets, as silencing some may not result in any observable phenotype or impact on pathogenicity (Govindarajulu et al., 2015; Guo et al., 2019). In this study, we confirmed that *BgtActin* is a suitable SIGS target, though further studies are needed to evaluate the general applicability of SIGS across different gene families and functional categories.

In the infection time-course analysis, *BgtActin* expression peaked at 15 hpi (**Figure 3**), corresponding with the timing of penetration. Previous studies have implicated actin cytoskeleton dynamics in appressorial morphogenesis, particularly via septin-mediated reorganization of F-actin and microtubules, and remodeling of the fungal cell wall (Ryder et al., 2022). Consistently, *BgtActin* silencing disrupted these critical developmental processes during the pre-penetration stages. Early application of dsRNA at 2 hpi reduced conidial germination and appressorium formation, while later application at 6 or 10 hpi led to the formation of abnormal appressoria, which were typically associated with defects in penetration peg differentiation. These observations support the conclusion that actin is indispensable for polarized growth and cellular morphogenesis during early infection in filamentous fungi (Walker and Garrill, 2006), and further highlight the sensitivity of the penetration stage to environmental or molecular interference (Gilbert et al., 2009; Zhang et al., 2022).

Finally, *BgtActin* silencing not only impaired early infection structure development but also led to a significant reduction in disease severity when dsRNA was applied at 6 or 10 hpi (**Figure 7**). In contrast, no significant reduction in disease symptoms was observed when dsRNA was applied at 2 hpi, reinforcing the importance of precise timing in SIGS application and supporting the view that *BgtActin* plays a key role during the penetration stage. Variance analysis further confirmed that both dsRNA treatment and application timing had significant effects on disease severity (**Table 2**), emphasizing the importance of temporal precision in SIGS-mediated gene functional studies.

## 5 Conclusion

In this study, the results provided direct evidence that both conidia and appressoria of *Bgt* are capable of exogenous dsRNA uptake. Notably, the efficiency of gene silencing was dependent on the timing of application. Leveraging this system, the critical role of *Actin* during the pre-penetration stages of *Bgt* was elucidated. Given the obligate biotrophic nature of *Bgt*, which has long hindered functional studies during pre-penetration stages, the SIGS approach described here offers a valuable tool for gene function analysis at pre-penetration stages. Nonetheless, whether SIGS can be broadly applied for the functional characterization of a wide range of genes during the pre-penetration stages remains an open question and warrants further investigation.

## Data Availability

The datasets presented in this study can be found in online repositories. The names of the repository/repositories and accession number(s) can be found in the article/**Supplementary Material**.

## Footnotes

1 Manuscript submitted to *Applied and Environmental Microbiology* on March 2025 (ID: AEM00700-25)
