# Supplementary material for: Spray-induced gene silencing enables the characterization of gene function during pre-penetration stages in Blumeria graminis f. sp. tritici
Source: Front Plant Sci. 2025 Jun 25;16:1628068. doi: 10.3389/fpls.2025.1628068 (PMC12239746; doi:10.3389/fpls.2025.1628068)
Supplement: Supplementary file 2 [file Image1.pdf]

Supplementary Figures

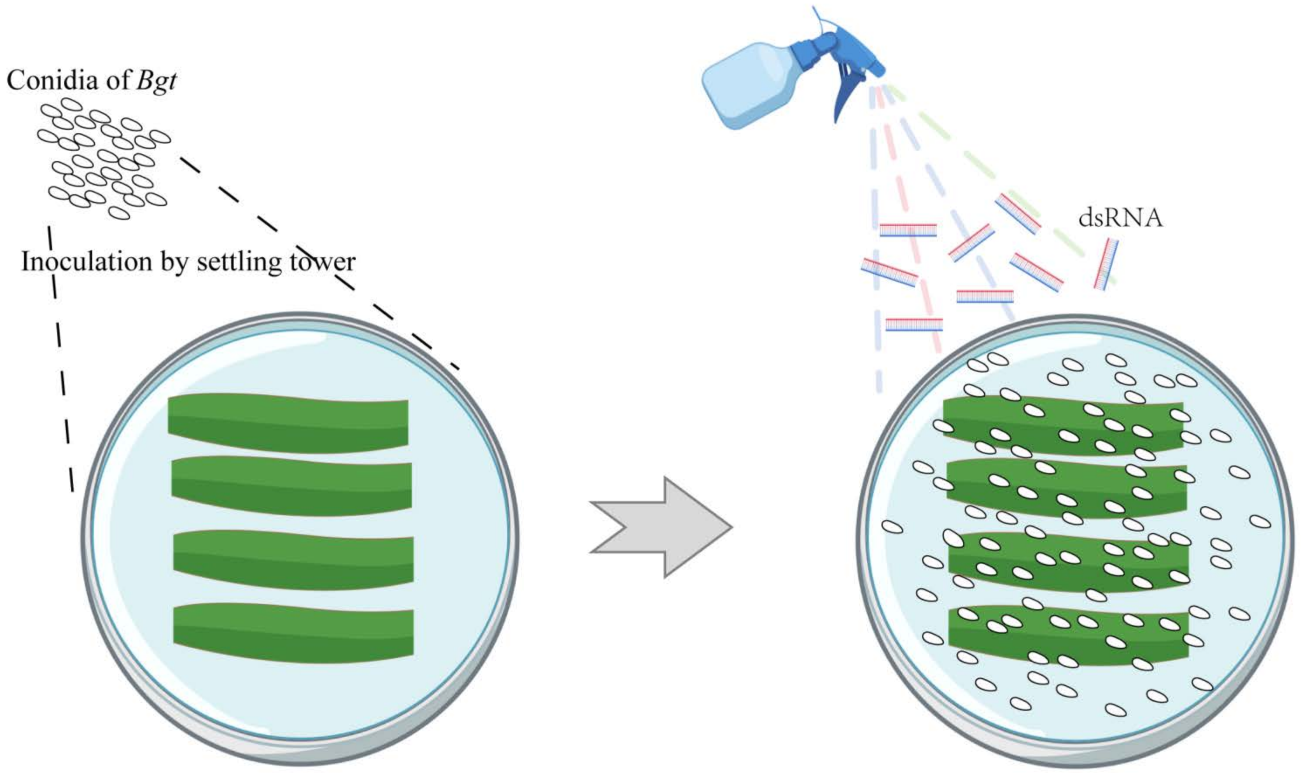

Supplementary Figure 1 Schematic depiction of double-stranded RNA spraying on *Blumeria graminis* f. sp. *tritici*.
